# Assessing vision in a baby

**Published:** 2016

**Authors:** Richard Bowman

**Affiliations:** Senior Lecturer: Public Health Ophthalmology, London School of Hygiene and Tropical Medicine, London, UK.

**Figure:**
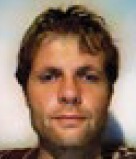
Richard Bowman

Don't be anxious about examining a baby. If the baby is awake and attentive, there is a lot you can find out by asking the parents and by simply observing the baby's reactions.

- First, ask the parents what they think about their baby's vision.
- Notice how the baby looks at things in the room, such as the window or any lights.
- Watch for eye contact between the baby and parents.
- Does the baby look when someone comes into the room?
- Does the baby respond to silent smiles orto raised eyebrows?
- Do you get eye contact?

You should have realistic expectations about what a baby should be able to do by a certain age. Table 1 shows when a baby is too young to show a visual response, when the response is likely to develop, and at what age you should be worried if a baby does NOT show the expected response. You can ask the mother or check the baby's responses yourself.

**Figure 1.**
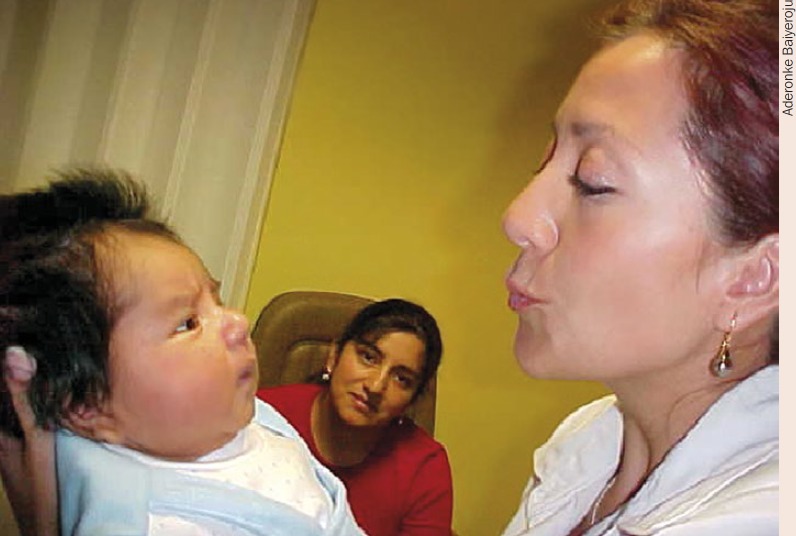
An eye care worker checks a baby's fixation. The baby is looking at her face, which is a reassuring sign.

For example, if a baby of about three weeks old does not turn to a diffuse light, such as light coming from a window, you would not necessarily be worried – although you would still believe the parents if they are concerned. On the other hand, if a baby is eight weeks old and does not eventually turn to a diffuse light, then there may be a problem and you should investigate further.

Bear in mind that there can be a lot of variation in babies' development; however, the table should be a helpful guide.

The most common and helpful test is the ability to fix and follow a light or a face.

**Table 1.** Normal visual functioning for a baby

Behaviour	Age				
	Neonate	6 weeks	3 months	4 months	5 months +
Blinks when a light is flashed in their eyes?	Healthy babies will do this. If not, suspect a problem				
Turns to a diffuse light, such a light coming from a window?	May do it	Healthy babies will do this. If not, suspect a problem			
Looks at your face when 10–20 cm away (less than 1 foot)? Any response to silent smiles or eyebrow raising?	Too young	May do it	Healthy babies will do this. If not, suspect a problem		
Eyes fix on, and follow, a dangling ball or toy?	Too young	May do it	Healthy babies will do this. If not, suspect a problem		
Watches an adult at 1.5 metres (5 feet)?	Too young	May do it		Healthy babies will do this. If not, suspecta problem	
Converges accurately? (If you move a toy closer and further away, do the eyes focus on the toy and line up properly?)	Too young	May do it		Healthy babies will do this. If not, suspecta problem	
Blinks in response to a threat? (Any silent, sudden movement close to the face which causes no breeze, e.g., opening your fist very suddenly.)	Too young	Too young	Too young	May do it	Healthy babies will do this. If not, suspect a problem

**Figure 2.**
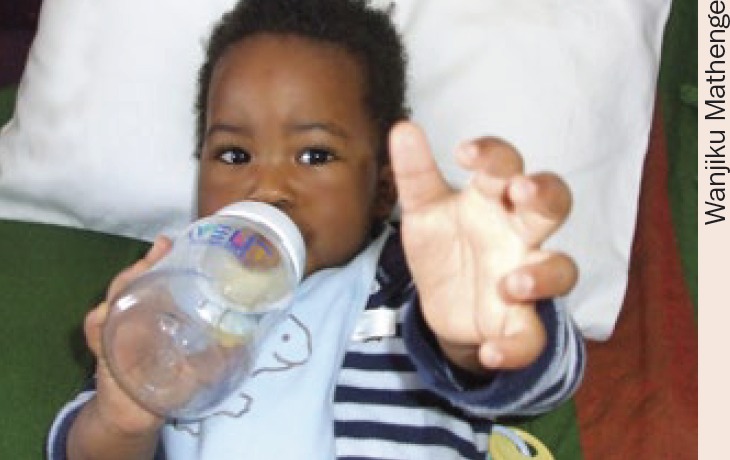
A healthy baby with good fixation. It is clear that he can see the camera and is reaching out for it.

This can be recorded using CSM notation, where:

- C = Central (fixing)
- S = Steady (following)
- M = Maintained (fixation resumed after cover/uncover)

## Tips for examining a baby

- Try to carry out as much of the examination as possible without touching the baby. Children often resist having their eyes held open, for example.
- Have many toys available. For each new toy, the baby will momentarily hold their eyes steady, allowing a quick examination. If available, use toys which are bright and can flash on and off. A good rule to remember is one toy, one look.
- Don't be embarrassed about making funny noises! These help to attract the baby's attention and to keep them interested and calm.
- In order to be able to do a more detailed examination in an infant, examine the child while he or she is being bottle fed or breast fed.

**Figure 3.**
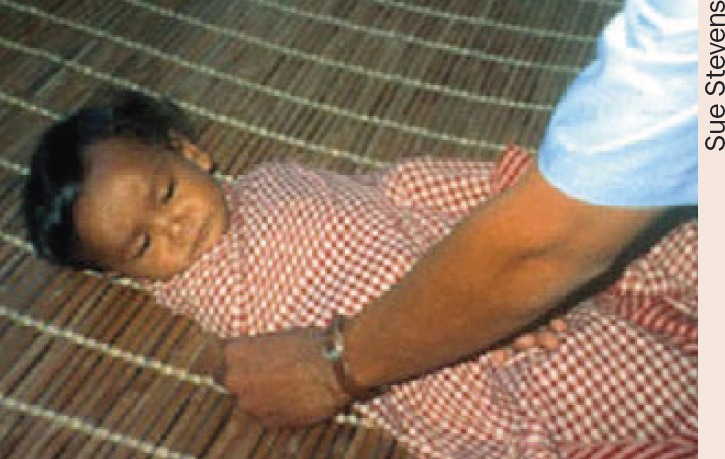
Wrapping a baby for an eye examination.

If you are struggling, ask the parent's permission to wrap the baby. Place the baby on a blanket or sheet, hold the arms to the side and the legs straight, and wrap the blanket around the body and arms (Figure 3). Ask the parent to hold the baby. Either the parent or a helper can then carefully open one eye at a time for the examination by gently holding the eyelids apart, without putting pressure on the eye. Remember that this may be very stressful for both the baby and the parent.

